# Quantitative pixel-wise measurement of myocardial blood flow: a comparison of methods to correct for surface coil-related field inhomogeneity

**DOI:** 10.1186/1532-429X-16-S1-P364

**Published:** 2014-01-16

**Authors:** Christopher A Miller, Li-Yueh Hsu, Allison D Ta, Hannah Conn, Susanne Winkler, Andrew E Arai

**Affiliations:** 1 Cardiovascular and Pulmonary Branch, National Heart, Lung and Blood Institute, National Institutes of Health, Bethesda, Maryland, USA

## Background

Pixel-wise quantitative analysis of cardiovascular magnetic resonance (CMR) perfusion images allows myocardial blood flow (MBF) to be measured at the level of approximately 30 μL of myocardium. However B1-field inhomogeneity, induced by phased array surface receiver coils, potentially confounds MBF measurements. The aim of this study was to compare the effect of: 1. no surface coil intensity correction (No-SCIC); 2. SCIC performed using saturation recovery steady-state free precession images (SSFP-SCIC); and 3. SCIC performed using proton density-weighted images (PD-SCIC); on pixel-wise measurements of MBF in patients with confirmed significant coronary artery disease (CAD) and healthy volunteers.

## Methods

26 subjects, comprising 18 patients (11 male; age 58 ± 12 years) with significant CAD (> 70% stenosis on invasive coronary angiography) in 1 (56%) or 2 (44%) major epicardial arteries (78% LAD, 50% RCA, 17% Cx) and 8 healthy volunteers (7 male; age 24 ± 10 years), were included. Perfusion imaging was performed using a motion-corrected saturation recovery steady-state free precession, dual sequence method at 1.5T (Espree, Siemens Medical Solutions) during Regadenoson vasodilator stress and at rest, using 0.05 mmol/kg Gadolinium-DTPA. A proton density-weighted image was acquired at the beginning of the perfusion sequence. B1-field inhomogeneity was approximated using a third-order surface fit to myocardial and body signal intensities on the PD image or the first SSFP image. The estimated intensity bias field was subsequently applied to the image series. For No-SCIC analysis, only baseline normalisation was performed. Pixel-wise MBF was measured from mid-ventricular stress images.

## Results

**Figure 1:**
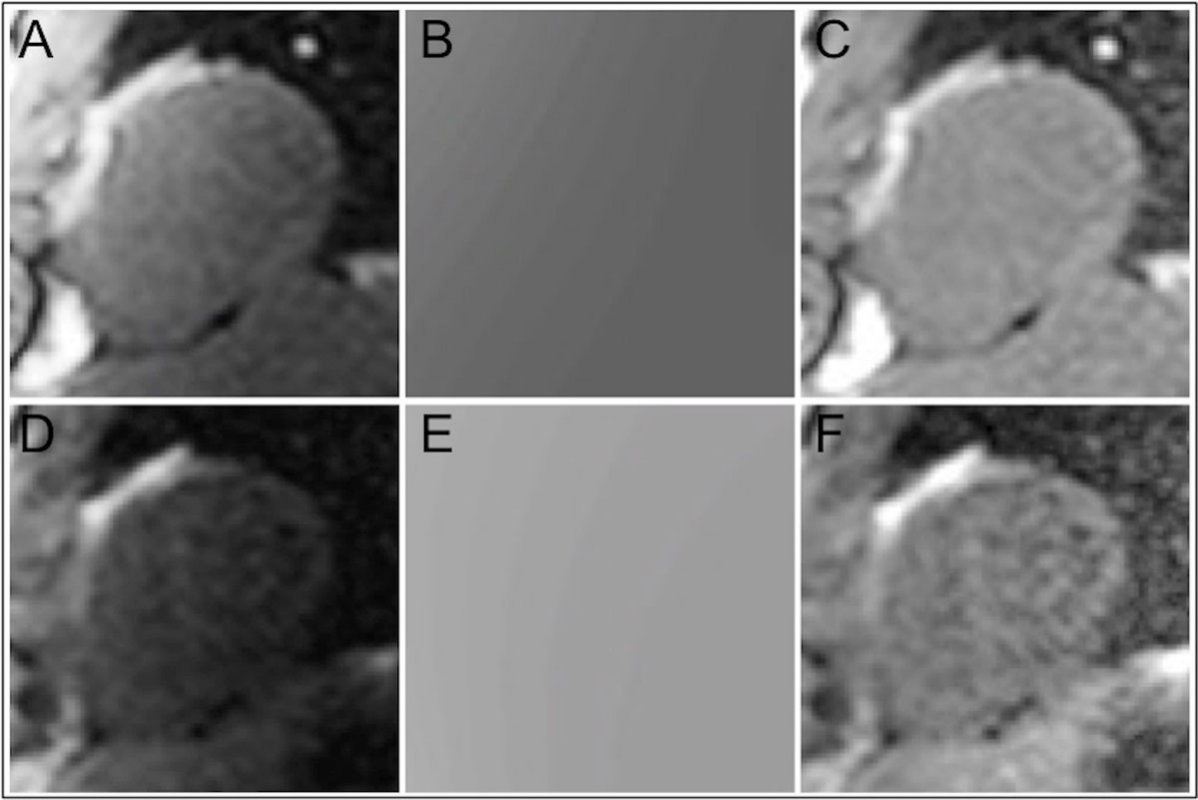
**Surface coil intensity correction (SCIC) using proton density-weighted images (PD-SCIC; A-C) and SSFP images (SSFP-SCIC; D-F)**. B1-field inhomogeneity was approximated using a third-order surface fit to myocardial and body signal intensities on PD (A) or SSFP (D) images in order to generate an intensity bias field (B and E), which was subsequently applied to the perfusion image series. The corrected PD and SSFP images are displayed in C and F respectively.

**Figure 2:**
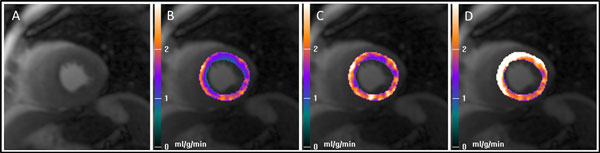
**Mid-ventricular stress image (A) and corresponding myocardial blood flow pixel maps (B-D) in a patient with a significant stenosis in the left anterior descending (LAD) artery**. The myocardial blood flow maps generated using surface coil intensity correction performed using a proton density-weighted image (PD-SCIC; B) and using a SSFP image (SSFP-SCIC; C) demonstrate a perfusion defect in the anterior wall and septum corresponding to the LAD stenosis. However, in the map generated without applying SCIC (No-SCIC; D), blood flow is highest in the anterior wall and septum, the opposite of what is correct.

There was significantly greater variation in signal intensity on the corrected SSFP-SCIC images (coefficient of variation 20 ± 7%) compared to the PD-SCIC images (10 ± 5%; p < 0.001, Figure 1). There was also significantly greater MBF spatial heterogeneity (standard deviation divided by mean) in healthy volunteers using SSFP-SCIC (24.8 ± 4.1%) compared to PD-SCIC (20.8 ± 3.0%; p = 0.009). Using No-SCIC, spatial heterogeneity was substantially higher (36.2 ± 6.3%; p = 0.001 compared with SSFP-SCIC; p < 0.001 compared with PD-SCIC). However, the difference in MBF between remote and ischaemic territories (using standard AHA/ACC segmentation) was not significantly different for SSFP-SCIC (0.63 ± 0.89 mL/min/kg) compared to PD-SCIC (0.50 ± 0.63 mL/min/kg; p = 0.145). Using No-SCIC, the difference in MBF between remote and ischaemic territories was minimal (0.06 ± 0.91 mL/min/kg), and significantly lower than for both SSFP-SCIC (p = 0.005) and PD-SCIC (p = 0.013; Figure 2).

## Conclusions

This study demonstrates the importance of correcting for surface coil-related field inhomogeneity for pixel-wise MBF quantification. However, although PD-based SCIC led to lower spatial heterogeneity than SSFP-based SCIC, the difference in measured MBF between ischaemic and remote territories was similar for both methods, and thus either could be applied.

## Funding

CAM is supported by the National Institute for Health Research, UK. LYH, AT, HC, SW and AEA are supported by the National Institutes of Health.

